# Lymphocytic Choriomeningitis Virus Infections in Hungary between 2017–2023—Investigation of the First Congenital Infections

**DOI:** 10.3390/diagnostics14131436

**Published:** 2024-07-05

**Authors:** Anita Koroknai, Anna Nagy, Orsolya Nagy, Nikolett Csonka, Eszter Mezei, Katalin Szomor, Mária Takács

**Affiliations:** 1 National Reference Laboratory for Viral Zoonoses, National Center for Public Health and Pharmacy, 1097 Budapest, Hungary; koroknai.anita@nngyk.gov.hu (A.K.);; 2 Institute of Medical Microbiology, Semmelweis University, 1089 Budapest, Hungary; 3 Department of Epidemiological and Vaccination Surveillance, National Center for Public Health and Pharmacy, 1097 Budapest, Hungary

**Keywords:** lymphocytic choriomeningitis virus, robovirus, congenital infection, meningitis, developmental disorders

## Abstract

Lymphocytic choriomeningitis virus (LCMV) is a neglected rodent-borne arenavirus, primarily spread by common house mouse species. Acquired human infections range from asymptomatic to mild flu-like symptoms and self-resolving neurological diseases. In contrast, intrauterine LCMV infection is associated with high mortality and morbidity. Infection of the fetus often leads to fetal death, and surviving fetuses may develop vision impairment and central nervous system developmental disorders. LCMV is mainly diagnosed by serological methods using in-house indirect immunofluorescence assays. LCMV nucleic acid is detected by the nested RT-PCR method and confirmed by Sanger sequencing. In Hungary, 23 acquired lymphocytic choriomeningitis cases were diagnosed between 2017 and 2023. Ten out of 23 confirmed patients proved to be positive by the PCR method. Two cases of intrauterine LCMV infections were detected in 2019 and 2021, respectively. The IgG antibody titers measured in the infant’s serum samples were much higher than the IgG titers of the maternal serum samples. Both IgM and IgA antibodies were detectable in the infants’ sera. As the microbiological diagnosis of LCMV is rather challenging and the symptoms are very similar to the clinical picture of other common teratogenic pathogens such as cytomegalovirus or *Toxoplasma gondii*, intrauterine LCMV infections might still be underdiagnosed.

## 1. Introduction

Lymphocytic choriomeningitis virus (LCMV; *Mammarenavirus choriomeningitidis*) is an enveloped, single-stranded RNA virus with a bisegmented genome. The small RNA segment (S) encodes the viral nucleoprotein and the glycoprotein precursor, which is cleaved into two subunits (GP1 and GP2). The large RNA segment (L) encodes the RNA-dependent RNA polymerase (RdRp) and a small zinc-finger protein, Z-protein [1]. Four genetic lineages (I-IV) of the LCMV are distinguished based on the S segment sequences, and three (I-III) based on the L segment sequences [2,3]. LCMV is the prototypic member of the *Arenaviridae* family and was discovered in 1933 by Armstrong and Lillie as a causative agent of aseptic meningitis [4]. House mouse species (*Mus musculus musculus*, *Mus musculus domesticus*) are the natural hosts and principal reservoirs of the LCMV. The virus can also be found in bank voles, pet rodents, rats, and guinea pigs [5,6]. Several outbreaks caused by LCMV have been linked to exposure to infected hamsters [7,8,9]. According to the distribution of mice, LCMV is found worldwide (except for Antarctica) but seems to be predominant in North America and Europe. Infections can occur at any time of the year, but most LCMV infections are reported during late autumn and early winter, when rodents move into human homes from the cold [10]. Asymptomatically infected animals excrete large amounts of the virus in their feces, urine, saliva, and nasal secretions during their lifetime. Human infections occur via direct or indirect contact with the excreta of infected rodents: inhaling the aerosolized virus, consuming contaminated food, or by rodent bites [5,11]. Approximately one-third of acquired infections are asymptomatic, or only mild, non-specific febrile disease develops. However, neurological diseases such as aseptic meningitis, encephalitis, or meningoencephalitis can occur in more severe cases [3,10]. Postnatal LCMV infection is usually non-fatal, and patients recover completely in most cases. The mortality rate is less than 1% [3].

Exposure to the virus during pregnancy can lead to intrauterine infection of the fetus. Infection in early pregnancy can induce spontaneous abortion and fetal death, and the surviving fetuses will develop mostly neurological and visual impairments. Chorioretinitis, hydrocephalus, and psychomotor retardation are the predominant characteristics of congenital LCMV infections. In addition, ventriculomegaly, periventricular calcifications, and microcephaly are also often demonstrated [3,5,10,12]. Systemic manifestations rarely develop as the virus exhibits a strong neurotropism [13]. The mortality rate among infants diagnosed with congenital LCMV infection is approximately 35%, and the survivors show long-term neurologic sequelae [14]. Human-to-human horizontal transmission has not been well documented; however, virus transmission via organ transplantation has already been reported [15,16,17,18].

In this study, we summarize the acquired human LCM virus infections in Hungary over the last six years and present two cases of serologically confirmed congenital LCMV infections. Hungary’s first congenital LCMV infection was reported in 2020 ([19], in Hungarian). The National Reference Laboratory for Viral Zoonoses (National Center for Public Health and Pharmacy, Budapest, Hungary) is exclusively responsible for the laboratory diagnosis of human LCMV cases in the country. Our work describes and represents the nationwide epidemiological and microbiological data of human LCMV infections.

## 2. Materials and Methods

During 2017–2023, the Hungarian National Reference Laboratory for Viral Zoonoses received 3557 test requests in regard to 1924 patients for LCMV with non-specific symptoms such as headache, fever, nausea, vomiting, dizziness, and neurological symptoms like vertigo, photophobia, somnolence, nuchal rigidity, confusion, paraparesis, and cerebrospinal fluid deviations. In the case of 23 patients, acute or recently acquired LCMV infection was confirmed based on the serological results. All LCMV confirmed patients had neurological signs in addition to non-specific symptoms. Except for one patient from the capital, all patients lived in rural areas. As we know, none of the confirmed patients were immunocompromised.

The first intrauterine LCMV infection was detected in Hungary in October 2019 [19]. Since then, special attention has been paid to samples submitted to the Virology Department of the National Center for Public Health and Pharmacy for investigation of congenital and ultrasound abnormalities. Clinicians usually request testing for cytomegalovirus, herpes simplex, rubella, or Zika virus, but not for LCMV. Within the framework of a prospective study, clinical specimens of 48 patients were examined for intrauterine LCMV infection between October 2019 and 2023 at the Hungarian National Reference Laboratory for Viral Zoonoses. Altogether, 37 infants with congenital neurological abnormalities and 11 pregnant women with ultrasound deviations were tested.

Fetuses, newborns, or infants with neurological disorders were investigated when they met at least one of the following 4 clinical criteria:

- Microcephaly, macrocephaly, or hydrocephalus;
- Periventricular calcifications;
- Chorioretinitis;
- Other central nervous system disorders.

A virus-specific immune response was detected in human sera and cerebrospinal fluid (CSF) samples using an in-house indirect immunofluorescent assay (IFA). In most cases, both serum and CSF samples were available and examined from the same patient. If LCMV-specific IgG antibodies were detected, IgG antibody titers and IgM and IgA antibody responses were also measured. To avoid the false interpretation, in the case of early sampling, a second serum sample was also collected from the same patient, on average 2–3 weeks later, compared to the first sampling. In-house IFA was carried out as described previously in the literature [20], with minor modifications.

The National Reference Laboratory for Viral Zoonoses evaluates acute or recently acquired LCMV infections based on the following serological results (at least one of the following criteria must be met):

- Detection of LCMV-specific IgG antibodies in high titers in the serum sample, in addition to the presence of IgM antibodies.
- Seroconversion or a four-fold increase in the LCMV-specific antibody titers in paired sera.
- Detection of LCMV-specific IgM antibodies in CSF samples.

Intrauterine LCMV infection was confirmed by the following laboratory results:

- Detection of LCMV-specific IgM or IgA antibodies in the infant’s sample.
- Detection of at least four-fold higher anti-LCMV IgG titers in the infant’s serum sample, compared to the IgG titer of the maternal serum.

For the detection of LCMV RNA, 20 serum, 13 CSF, and 9 urine samples of serologically confirmed patients were used. Samples were stored at −80 °C until molecular testing. Total nucleic acid was extracted from 140 µL of the specimens using a QIAmp Viral RNA Mini Kit (QIAGEN, Hilden, Germany) according to the manufacturer’s instructions. Five µL of extracted RNA was reverse transcribed in a 20.5 µL final reaction volume. The reverse transcription reaction mixture consisted of the following reagents: 3 µL of Ultra Pure DNase/RNase-free distilled water (Invitrogen^TM^, Thermo Fisher Scientific, Waltham, MA, USA), 2 µL of 10× MuLV Reverse Transcriptase Buffer (New England BioLabs, Ipswich, MA, USA), 4 µL of 25 mM MgCl_2_ (Thermo Fisher Scientific, Waltham, MA, USA), 4 µL of dNTP mix (10 mM each) (Applied Biosystem, Thermo Fisher Scientific, Waltham, MA, USA), 1 µL of 50 µM random hexamer primers (Invitrogen^TM^, Thermo Fisher Scientific, Waltham, MA, USA), 0.5 µL of 200 U/µL MuLV Reverse Transcriptase (New England BioLabs, Ipswich, MA, USA) and 1 µL of 20 U/µL RNase Inhibitor (Applied Biosystem, Thermo Fisher Scientific, Waltham, MA, USA). The reverse transcription reaction was incubated at 25 °C for 5 min and 42 °C for 30 min, and enzyme inactivation was carried out at 99 °C for 5 min, followed by rapid cooling to 4 °C.

RT-PCR products were amplified using a nested or semi-nested PCR protocol with the following primer sets (Table 1): A 165-nt region in the S segment, targeting the nucleocapsid gene, was amplified by using primers described previously by Park et al. with minor modifications (outer primers: NP16 and NP17-1; internal primers: NP21-1 and modified NP20-1) [21]. In 2020, a new PCR protocol was introduced that uses a primer set targeting the RdRp gene of the L segment, resulting in a longer (443 nucleotide-long) PCR amplicon (outer primers: LCML3160-plus and LCML3722-minus, internal primers: LCML3160-plus and LVL3754-minus) [22]. The amplification reaction mixtures of both the first- and second-round PCR assays consisted of 5 µL of template DNA, 5.5 µL of Ultra Pure DNase/RNase-free distilled water (Invitrogen^TM^, Thermo Fisher Scientific, Waltham, MA, USA), 12.5 µL of ready-to-use 2× My Taq Red Mix (Bioline, Meridian Bioscience, Cincinnati, OH, USA), and 2.0 pmol of each primer. PCR products were visualized in a 2% Tris-borate-EDTA agarose gel stained with ECO Safe (Pacific Image Electronics, Torrance, CA, USA).

To perform Sanger sequencing, nested PCR amplicons were purified using Viogene’s Advanced^TM^ PCR Clean Up System (Viogen Biotek Corporation, New Taipei City, Taiwan) following the manufacturer’s instructions. Direct sequencing of the amplicons was performed on a 3500 Genetic Analyzer (Applied Biosystems, Thermo Fisher Scientific, Waltham, MA, USA) using the BigDye^®^ terminator V3.1 cycle sequencing kit (Applied Biosystems, Thermo Fisher Scientific, Waltham, MA, USA) according to the manufacturer’s recommendations. Bidirectional sequencing of the DNA region of interest was performed using the following primers: NP-21-1 and modified NP-20-1 primers for the S segment, and LCML3160-plus and LVL3754-minus primers for the L segment, respectively. Nucleotide sequences were identified using the Basic Local Alignment Search Tool (BLAST, http://blast.ncbi.nlm.nih.gov/Blast.cgi; accessed on 15 January 2024). A phylogenetic maximum likelihood tree was created by MEGA 11 (Molecular Evolutionary Genetic Analysis) software (version 11) using ClustalW alignments of a 390 nucleotide-long partial sequence of the L segment of LCMV. Bootstrap analysis of 1000 replicates was carried out to estimate the tree topology’s reliability. The evolutionary distance was calculated using the General Time Reversible model.

## 3. Results

**Figure 1:**
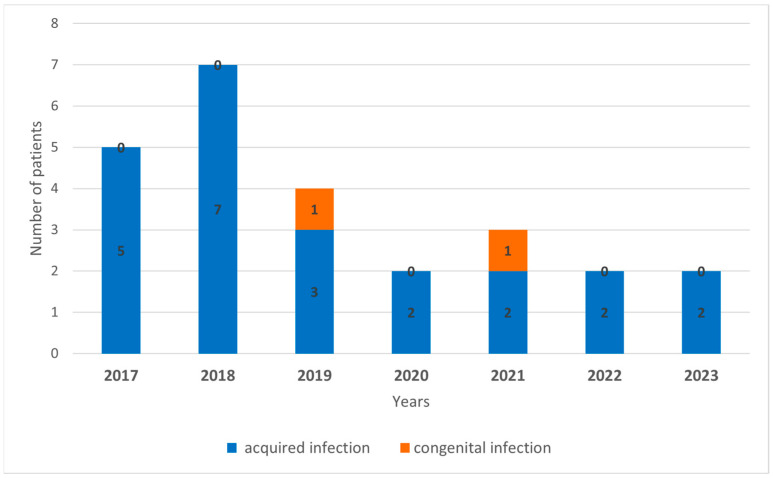
The annual number of laboratory-diagnosed LCMV cases in Hungary between 2017 and 2023.

**Figure 2:**
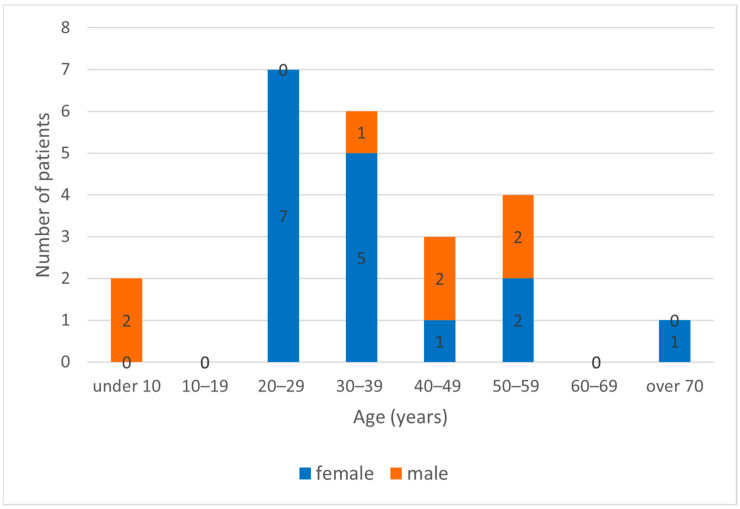
Age and gender distribution of acquired LCMV-infected patients in Hungary between 2017 and 2023.

Between 2017 and 2023, 23 cases of acquired LCMV infection and two cases of intrauterine LCMV infection were diagnosed by serological methods in Hungary. The number of annual cases is shown in Figure 1. The average age of LCMV-infected patients was 35.5 (5–73) years. LCMV infections mostly occurred in females; 69.6% (16 patients) of the confirmed cases were female, and 30.4% (7 patients) were male (Figure 2).

Ten out of 23 serologically confirmed patients were found to be positive by the PCR method. LCMV RNA was detected in four serums, five CSF samples, and one urine sample. The anamnestic data and serological and molecular results of LCMV PCR-positive patients are summarized in Table 2. In the cases of patient 1 and patients 3–7, primers designed for the S segment were used for PCR testing, and a partial nucleocapsid gene was amplified. In patients 8–10, primers targeting the L segment were applied, and a partial RNA polymerase gene was amplified. In patient 2, the S segment-specific PCR was negative. In contrast, L segment-specific PCR was retrospectively performed, and this method found the re-tested sample to be PCR positive. Re-testing of samples of patient 1 and patients 3–7 by L segment-specific PCR was not possible, as the amount of properly stored samples was not sufficient. The six short partial sequences of the S segment nucleocapsid gene (of patient 1 and patients 3–7) are shown in the Supplementary Data. The urine sample of patient 9 could only be sequenced in one direction; therefore, this shorter RdRp sequence is not aligned with the phylogenetic tree. This sequence can also be found in the Supplementary Data.

**Figure 3:**
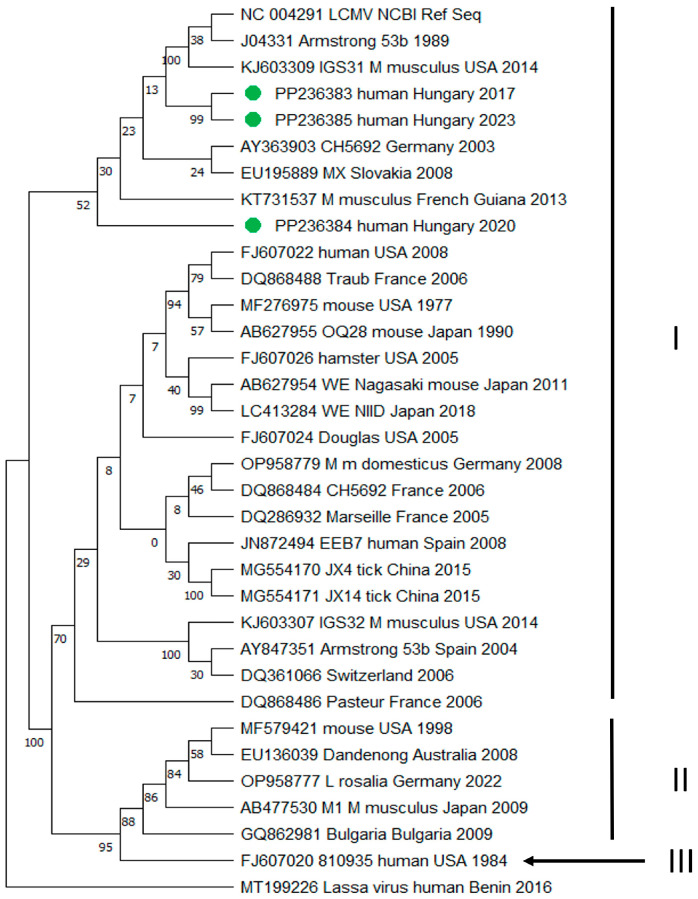
Maximum likelihood phylogenetic tree of LCMV sequences based on the 390 nucleotide-long region of the L segment RNA polymerase (RdRp) gene. The Lassa virus was used as an outgroup. Sequences obtained from CSF samples of three Hungarian patients are indicated by green circles (GenBank accession numbers: PP236383, PP236384, and PP236385). LCMV sequences were named as follows: GenBank accession number, strain name, host species, country of origin, and the year of the isolation/submission (if data were available). Roman numerals (I–III) represent the different LCMV lineages defined according to Albariño et al. [2].

Phylogenetic analysis based on the 390-nucleotide-long region of the L segment RNA polymerase gene obtained from CSF samples of patients 2, 8, and 10 revealed a close phylogenetic relationship between the Hungarian samples and virus strains from Germany, USA, Slovakia, French Guiana, and the Armstrong 53b strain (GenBank accession numbers: AY363903, KJ603309, EU195889, KT731537, J04331) (Figure 3). Based on the sequence homologies, all samples belonged to LCMV lineage I; however, they form a separate branch within the phylogenetic tree. Our sequences are available under GenBank accession numbers PP236383-85. Two sequences (PP236383 and PP236385) were placed on one branch within the phylogenetic tree, and the sequence of PP236384 was separated. There was only one amino acid difference between PP236383 and PP236385 (99.23% homology), and there was 88.97% homology at the nucleic acid level. Between PP236383 and PP236384, there was 87.69% homology at the amino acid level and 80.77% homology at the nucleic acid level. The amino acid sequence homology of PP236384 and PP236385 was 86.92%, while at the nucleic acid level, it was 77.44%.

The first intrauterine LCMV infection in Hungary was diagnosed in a 6-week-old premature baby with microcephaly and chorioretinitis in October 2019 (Case 1) [19]. In March 2021, congenital LCMV infection was confirmed in another case in a 6-month-old microcephalic infant (Case 2). Table 3 summarizes the most important anamnestic data and serological results of congenital LCMV cases. In both cases, TORCH (*Toxoplasma gondii*; rubella virus; cytomegalovirus, CMV; herpes simplex virus, HSV) serology did not confirm intrauterine infection. Samples of both patients together with the maternal sera were further tested for LCMV-specific antibody responses. In comparison with the maternal sera, the LCMV-specific IgG titer of the patients was more than four-fold higher than the LCMV-specific IgG titer measured in maternal samples. In Case 1, both IgM and IgA antibodies were detectable; however, S segment-specific RT-PCR was negative from serum, whole blood, and urine samples as well. Amniotic fluid, cerebrospinal fluid, and blood samples at birth were not available. According to the patient history, the mother experienced a self-resolving illness with flu-like symptoms during the first trimester of the pregnancy. In addition, the family keeps poultry at home, and there might be rodents around the poultry farm. In Case 2, no additional anamnestic and epidemiologic data were available.

## 4. Discussion

In Hungary, viral encephalitis and meningitis have been notifiable infectious diseases since 1950. Therefore, the data on diseases with such diagnoses are collected as a framework for syndrome-based surveillance. Diseases reported as viral encephalitis and meningitis also include diseases caused by LCMV.

**Figure 4:**
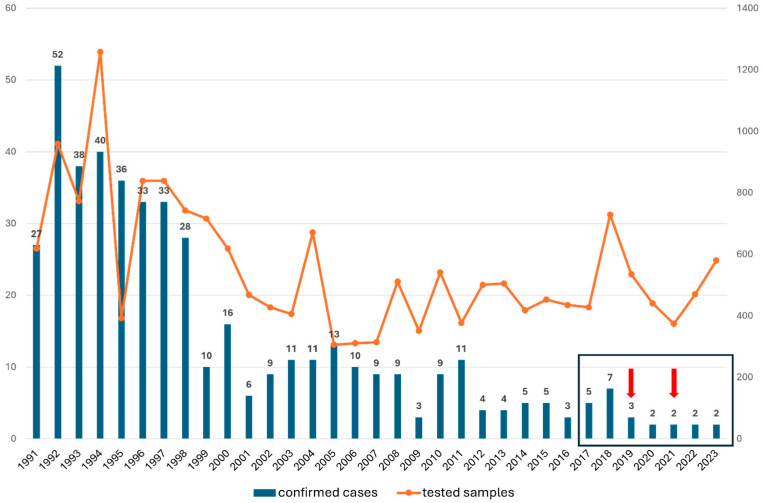
The annual number of laboratory-diagnosed LCMV cases and the number of clinical samples tested for LCMV in Hungary, between 1991 and 2023. (Based on the data of the National Center for Public Health and Pharmacy, Budapest, Hungary) The framed part represents the data of the period discussed in this study. Red arrows indicate the years when the two congenital cases were diagnosed.

In the last six years, lymphocytic choriomeningitis has been rarely diagnosed in Hungary. However, a 1973–1977 Hungarian hospital study [23] showed that 8.8% (143/1630) of meningitis cases were associated with LCMV infection, and 23–41 cases were diagnosed annually. A total of 48.2% (69 patients) of the positive cases were male, and 51.8% (74 patients) were female. According to the National Center for Public Health and Pharmacy, Budapest, Hungary, data, the number of confirmed cases between 1991 and 1998 was also significantly higher (27–52 cases/year) than the average annual case numbers between 2017 and 2023. The tendency decreased to 6–16 cases in the 2000s, while from 2012 only 3 to 5 cases were diagnosed yearly. According to the annual number of samples tested for LCMV between 1991 and 2023, the number of tested samples was higher between 1990 and 2000 (an average of 775 samples were tested annually) than in the following years. Between 2001 and 2011, the annual average was 425 tests, while from 2012 to 2023, the number of tested samples increased slightly (the annual average was 489 tests) (Figure 4). Despite these facts, only a few LCMV-positive cases were diagnosed annually during the last period between 2012 and 2023.

However, there are some possible explanations for the decreasing tendency of laboratory-confirmed cases. It is important to note, that neither human nor rodent seroprevalence data are currently available in Hungary. We can assume that most of the LCMV infections may be underdiagnosed. This theory is supported by the high proportion of asymptomatic infections or infections with milder, self-limiting symptoms when the patient’s condition does not require medical care; therefore, no laboratory investigation is carried out either.

The literature data also shows that lymphocytic choriomeningitis is underdiagnosed [3,10]. One of the possible reasons could be the limited number of commercially available test kits. Special laboratory conditions are required for virus propagation and the production of in-house diagnostics, such as IFA. Based on the ECDC website, LCMV-specific serological methods are available in only 12 laboratories in Europe, while molecular diagnostic tests are performed in only 18 laboratories [24].

In most of the studies, LCMV seroprevalence in the human population was measured between 1 and 10% [13,25,26]. The proportion of seropositive people in Baltimore, USA, was approximately 5% [27,28], and in Nova Scotia, Canada, was 4% [29]. In urban areas of Argentina, the prevalence of LCMV-specific antibodies in humans was 1–3.6% compared to the 12.9% prevalence of house mice [30]. According to a 2003 study, the prevalence of LCMV in Spain was 1.7% in humans and 9% in wild rodents [31]. However, a much higher seroprevalence rate was reported in Bratislava, Slovakia (37.5%), and Vir Island, Croatia (36%) [32,33]. Several studies investigated the LCMV seroprevalence in risk groups such as forestry workers, rodent breeders, etc. The seropositivity proportion was 13% among zoo workers in Vienna, Austria [34]; 32% of the tested workers were found to be seropositive in a study performed at three breeding facilities in Kentucky, USA [35]. When investigating the samples of forestry workers in Italy, LCMV-specific antibodies were detected in 2.5% of the samples, compared to 5.6% of the rodent samples [36]. Seroprevalence studies in patients with neuroinvasive diseases in Finland found 5% seropositivity [37]. In another study from Iraq, the prevalence of LCMV was 7% in acute febrile patients and 12.2% in the healthy control group [38]. Among pregnant women, LCMV seropositivity was measured at 1.6% in Argentina and 3.9% in Croatia, respectively [30,39].

The exact prevalence of intrauterine LCMV infections is also still unknown. Since the first description by Komrower et al. in England in 1955 [40], there have been 27 reports about 86 congenital LCMV cases to date. Most of the congenital cases were reported in the United States [12]. According to the study of Ferenc et al., chorioretinitis is the most common symptom in congenital LCMV infections (present in 83.53% of the patients), and microcephaly is also often diagnosed (present in 38.82% of the patients) [12]. The diagnosis is mainly based on detecting LCMV-specific antibodies in fetal and maternal serum samples [41]. In the case of a congenital infection, the IgG titer measured in the infant’s serum samples is typically at least four-fold higher than the IgG titer measured in the maternal sample. The IgM response is rarely detectable, as most infections with developmental consequences may occur in early pregnancy [6,25]. Nucleic acid detection for the diagnosis of both prenatal and postnatal LCMV infections has limitations because of the short viremic period of the virus. Due to mild or asymptomatic infections, viral RNA is usually no longer detectable at the time of clinical suspicion of LCMV infection. Serum or CSF samples taken during the acute febrile phase of the disease may be the most suitable clinical materials for PCR testing. According to our results, viral RNA can most likely be detected in CSF during the meningitis phase. Another difficulty is the variability of the virus, which makes the design of suitable primers challenging. The time of the viral clearance from the infected fetus is unknown. Thus, it is possible that PCR can no longer detect the viral RNA from tissues or blood samples of the intrauterine-infected newborn at the time of birth [10]. However, LCMV-specific RNA was detected during pregnancy in amniotic fluid in two cases [42,43].

The differential diagnosis of congenital LCMV infections is complicated because other pathogens and genetic abnormalities (such as Aicardi-Goutieres syndrome, pseudo-TORCH syndrome, etc.) [44,45], can cause similar symptoms [10,46]. TORCH pathogens—the most common causative agents of developmental disorders—represent the first-line microbiological examination. Most commonly, congenital toxoplasmosis and CMV infection mimic the symptoms of congenital LCMV infections, since all three pathogens can cause chorioretinitis and microcephaly [14]. In contrast with classic TORCH infections, congenital LCMV is rarely associated with systemic anomalies and other organ manifestations, such as hepatosplenomegaly, anemia, thrombocytopenia, and hearing loss [5,6,11].

Since there is no specific therapy for the disease and only supportive treatment is available, preventive measures are essential. Pregnant women should be warned to avoid any contact with rodents during pregnancy. It is critical to minimize dust aerosolization in places frequently visited by rodents (for instance, warehouses, storage rooms, attics, basements, weekend houses, etc.). To prevent the inhalation of rodent excreta during cleaning, wearing personal protective equipment, such as face masks and gloves, is recommended. At the beginning of the process, it is important to avoid stirring up rodent excreta and nesting materials by sweeping or vacuuming. Cross-ventilation of the space is also important, as is using disinfectants to remove rodent urine or feces and disinfect the surfaces. Contamination of food and crops can be prevented by storing them in closed containers.

Intrauterine LCMV infections are probably underdiagnosed. However, LCMV is a neglected human pathogenic viral zoonosis. Our study highlights that LCMV infections could be laboratory-confirmed as a causative agent of congenital neurological and ophthalmic abnormalities in many more cases. The possibility of intrauterine LCMV infection should be raised in all fetal neurologic abnormalities and chorioretinitis with unknown etiology. This is especially true in cases where the mother had flu-like symptoms during pregnancy and if rodent contact of the pregnant woman cannot be excluded.

## 5. Conclusions

LCMV is a neglected viral disease, as a causative agent of meningitis or developmental disorders. It would be important to draw the attention of physicians, nurses, and midwives to LCMV as a potential teratogenic agent.

## Figures and Tables

**Table 1:** Oligonucleotide primers used in this study.

Primer Designation	Sequences 5′-3′
S segment nested RT-PCR	
NP16	CGCACAGTGGATCCTAGGC
NP17-1	GCTGACYTCAGARAAGTCCAACC
NP21-1	CCTAGGCATTTGATTGCGC
modified NP20-1	A**R**AAGRYT**R**GTTGCRTC**Y**TT *
L segment semi-nested RT-PCR	
LCML3160-plus	GCAAAGCTTGAATTTCAARTTTGA
LCML3722-minus	CTCGACAAGTTTATGCATRTGCCA
LVL3754-minus	CACATCATTGGTCCCCATTTACTRTGATC

* The bases marked in bold represent the modifications compared to the original sequences.

**Table 2:** Summary of the anamnestic data and serological and molecular results of LCMV PCR positive patients between 2017 and 2023.

Patients No.	Sample ID/Year	Specimen Type	Gender	Age (Year)	Symptoms	IgG (Titer)	IgM/ IgA	Amplified Partial Gene (Segment)	Genbank Accession Nr.
1	2915/2017	serum	Female	32	Meningitis (fever, headache, vomiting, nuchal rigidity)	≥1:640	+/+	nucleocapsid (S)	SD
2	3824/2017	CSF	Female	37	Meningitis (fever, headache, cerebral edema)	positive	+/+	RNA polymerase (L)	**PP236383**
3	4681/2017	serum	Male	52	Meningoencephalitis, paraparesis	≥1:160	+/+	nucleocapsid (S)	SD
4	75/2018	serum	Female	53	Meningitis (fever, headache, elevated CSF protein levels)	≥1:10	+/+	nucleocapsid (S)	SD
5	222/2018	serum	Female	23	Meningitis (fever, headache, vomiting, photophobia)	≥1:10	+/+	nucleocapsid (S)	SD
6	931/2018	CSF	Female	55	Meningoencephalitis (fever, headache, confusion)	positive	+/+	nucleocapsid (S)	SD
7	1689/2018	CSF	Female	43	Meningitis (fever, headache, nuchal rigidity)	positive	+/+	nucleocapsid (S)	SD
8	1539/2020	CSF	Male	5	Meningitis (fever, headache, vomiting, nuchal rigidity)	positive	+/+	RNA polymerase (L)	**PP236384**
9	3026/2022	urine	Female	38	Meningitis (fever, headache, nausea, vertigo, liver function abnormality)	n.t.	n.t.	RNA polymerase (L)	SD
**10**	**2576/2023**	CSF	Male	48	Meningoencephalitis (fever, headache, confusion)	positive	+/+	RNA polymerase (L)	**PP236385**

Note: Samples with a sequence aligned to a phylogenetic tree are marked in bold. CSF: cerebrospinal fluid; +/+: positive/positive; n.t.: not tested; SD: sequence is shown in the Supplementary Data.

**Table 3:** Summary of the anamnestic data and serological results of congenital LCMV infected infants in Hungary.

Case No.	Time of Diagnosis	Patient’s Age	Symptoms	Additional Anamnestic Data	Clinical Specimens	IgG (Titer)	IgM	IgA
1	October 2019	6-week-old premature baby	chorioretinitis, microcephaly, intrauterine dystrophy	TORCH and Zika serology did not confirm intrauterine infection. The mother had flu-like symptoms during the first trimester of the pregnancy. There were rodents around the house.	Newborn’s serum I.	≥1:640	borderline ^#^	positive
					Newborn’s serum II. (19 days later)	≥1:20,480	borderline ^#^	borderline ^#^
					Maternal serum	1:160	negative	borderline ^#^
2	March 2021	6-month-old child	microcephaly	Intrauterine toxoplasma infection was not confirmed from child’s serum I. Intrauterine rubella, CMV, and HSV infections were not confirmed from child’s serum II.	Infant’s serum I. (1.5 months old)	1:2560	negative	negative
					Infant’s serum II. (6 months old)	1:640	negative	negative
					Maternal serum I.	1:160	negative	negative
					Maternal serum II.	1:40	negative	negative

^#^ borderline means that only a few cells with positive morphology were detected.

## Data Availability

Data are contained within the article or Supplementary Materials.

## Supplementary Materials

The following supporting information can be downloaded at: https://www.mdpi.com/article/10.3390/diagnostics14131436/s1: Supplementary data: Detailed results of partial sequences of LCMV.
