# Supplementary material for: Lymphocytic Choriomeningitis Virus Infections in Hungary between 2017–2023—Investigation of the First Congenital Infections
Source: Diagnostics (Basel). 2024 Jul 5;14(13):1436. doi: 10.3390/diagnostics14131436 (PMC11241323; doi:10.3390/diagnostics14131436)
Supplement: Supplementary file 1 [file diagnostics-14-01436-s001.zip › diagnostics-3055498-supplementary.pdf]

Detailed results of partial sequences of LCMV

>2915/17_serum_S segment_155nt
GTTGCGTCYTTGATGACAACAGCCTTCACATCTGTTGTGAAGTTTWGCAGTTCCCTCCTCAGTGCCKGTGTCCA
CTGAAAGCTYTTAACTTCCTTGGACAGAGACATCTGTCCTCAATGAATCTCAAAGAAAAATGCGCAATCAAATG
CCTAGGA

>4681/17_serum_S segment_155nt
GTTGCGTCCTTGATGACAGCAGCCTTCACATCTGTTGTGAGTTTTGCAGTTCCCTCCTCAGTGCCKGTGTCCACT
GAAAGCTCTTAACTTCCTTGGACAGAGACATCTTGTTCCTCAATGAATCTCAAAGAAAAATGCGYAMTAAAYGC
CTAGGA

>75/18_serum_S segment_135nt
GGCTCATCATCTGTTGTGAGTTTTGCAGTTCCCTCCTCAGTGCCTGTGTCCACTGAAAGCTCTTGACTTCCTTGG
ACAGAGACATCTTGTTCCTTAATGAATCTCAAAGAAAAATGCGCAATCAAATGCCTAGGA

>222/18_serum_S segment_136nt
GTTGCGTCCTTGATGACAGCAGCCTTCACATCTGTTGTGAAGTTTTGCAGTTCCCTCCTCAGTGCCTGTGTCCAS
TGAAAGCTTTTGACTTCCTTGGACAGAGACATCTTGTTCCTTAATGAATCTCAAAGAAAAA

>931/19_CSF_S segment_153nt
GTTGCGTCYTTGATGACAGCGGCCTTGACATTCGAGGTAAAGCCCTGCAGCTCTCTCCTCAGTGCCTGAGTCCA
TTGGAAGCTTTTGACCTCTTTGGACAGAGACATTATAGGTCTCACAAGCACAAACAAAAGCGCAATCAAATGCC
TAGGA

>1689/19_CSF_S segment_158nt
GTTGCGTCYTTGATGACACTAGCCTTGACATTAGAGGTGAAGCCCTGCAGCTCTCTTCTCAATGCCTGAGTCCA
TTGGAAACTCTTGACTTCCTTGGACAAGGACATAGTGATGTCTTTCACTGTGTCTCCAACAAATGCGCAATCAA
ATGCCTAGGA
